# Crapemyrtle Bark Scale: A New Threat for Crapemyrtles, a Popular Landscape Plant in the U.S.

**DOI:** 10.3390/insects7040078

**Published:** 2016-12-16

**Authors:** Zinan Wang, Yan Chen, Mengmeng Gu, Erfan Vafaie, Michael Merchant, Rodrigo Diaz

**Affiliations:** 1 Department of Entomology, Agricultural Center, Louisiana State University, Baton Rouge, LA 70803, USA; wangzinan2014@gmail.com; 2 Hammond Research Station, Agricultural Center, Louisiana State University, Hammond, LA 70403, USA; YaChen@agcenter.lsu.edu; 3 Department of Horticultural Science, Texas A&M AgriLife Extension Service, College Station, TX 77843, USA; mgu@tamu.edu; 4 Department of Entomology, Texas A&M AgriLife Research & Extension Center, Overton, TX 75684, USA; Erfan.Vafaie@ag.tamu.edu; 5 Department of Entomology, Texas A&M AgriLife Research & Extension Center, Dallas, TX 75252, USA; m-merchant@tamu.edu

**Keywords:** *Acanthococcus lagerstroemiae* (Kuwana), exotic species, integrated pest management, host resistance, biological control, parasitoids, *Chilocorus cacti* L., *Hyperaspis* spp.

## Abstract

Crapemyrtle bark scale, *Acanthococcus* (=*Eriococcus*) *lagerstroemiae* (Kuwana) (Hemiptera: Eriococcidae), is a newly introduced insect pest on crapemyrtles, *Lagerstroemia* spp. (Myrtales: Lythraceae), one of the most popular flowering shrubs in the U.S. Since first detected in Texas in 2004, this pest has spread to twelve states causing losses to stakeholders. To develop a management plan, we reviewed current knowledge about the pest’s biology and ecology, and suggested research approaches including studying its thermal tolerance, host range, plant resistance and biological control. Parasitoids and predators have been reared from *A. lagerstroemiae* in the U.S. and China. However, new surveys of natural enemies should be conducted in China, and studies on the host range and impacts of natural enemies on *A. lagerstroemiae* may help determine the potential for classical biological control. The life history, preying efficiency and rearing methods are important for coccinellid predators found in the U.S. including *Chilocorus cacti* L. and *Hyperaspis* spp. To enhance natural enemy performance, it is important to evaluate a sustainable insecticide program that considers efficacy, timing, rate and impact on pollinator health. Finally, an integrated management program of *A. lagerstroemiae* is discussed including planting resistant cultivars, using host specific natural enemies, and prudent use of insecticides.

## 1. Introduction

Crapemyrtles, *Lagerstroemia* spp. L. (Myrtales: Lythraceae), are popular flowering shrubs and small trees around the world. Native to Southeast Asia and Australia, including China, Japan, India, Australia and Oceania [1], crapemyrtles have been introduced into the U.S. as ornamentals for 180 years [2]. Crapemyrtles have become a dominant landscape tree in the southern U.S. with an annual wholesale value of approximately $66 million in 2014 [3]. Breeding programs over the last 35 years have produced superior varieties in a wide range of plant sizes and growing habits with improved flowering, new flower and foliage colors, ornamental bark, increased vigor and adaptability to a wide range of soil types [2,4]. In the U.S., crapemyrtle is hardy from USDA Plant Hardiness Zone 6 to 10 (temperature ranging from −23.3 °C to −1.1 °C), while its roots are believed to be winter hardy in Zone 5 (temperature ranging from −28.9 °C to −23.3 °C) [2].

Crapemyrtles are valued for their relatively easy maintenance and limited pest problems [2,4]. The main diseases of crapemyrtle are powdery mildew caused by the fungus *Erysiphe australiana* (*=lagerstroemiae*) (McAlpine) U. Braun & S. Takamatsu (Erysiphales: Erysiphaceae), and Cercospora leaf spot caused by *Pseudocercospora lythracearum* (Heald & Wolf) Liu & Guo (Capnodiales: Mycosphaerellaceae) [2]. Until the discovery of *Acanthococcus lagerstroemiae* (Kuwana) (Hemiptera: Eriococcidae), commonly referred to as the crapemyrtle bark scale, the primary insect pests of crapemyrtle were the crapemyrtle aphid, *Sarucallis* (=*Tinocallis*) *kahawaluokalani* (Kirkaldy) (Hemiptera: Aphididae) and the Japanese beetle, *Popillia japonica* Newman (Coleoptera: Scarabaeidae), followed by flea beetles, *Altica* spp. Geoffroy (Coleoptera: Chrysomelidae), and the granulate ambrosia beetle, *Xylosandrus crassiusculus* (Motschulsky) (Coleoptera: Curculionidae) [2]. However, these pests on crapemyrtles can largely be managed with resistant cultivars, landscape planning including plant placement in sunny locations with good ventilation; monitoring programs with optimal traps and rapid response including trunk sprays or the removal of infested plants; and environmentally friendly insecticides including insecticidal soaps or horticultural oils [2,4,5].

**Figure 1:**
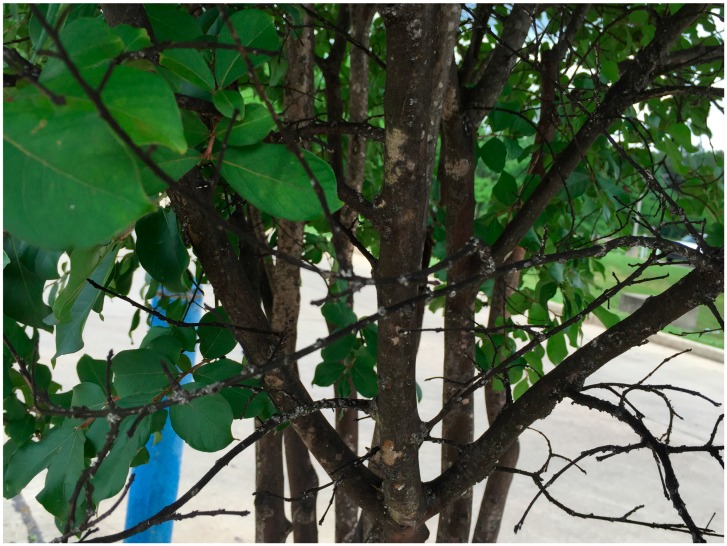
Branch dieback and accumulation of black sooty mold on the crapemyrtle tree infested with *Acanthococcus lagerstroemiae*.

The crapemyrtle bark scale, *A. lagerstroemiae*, is a newly introduced insect pest of crapemyrtles in the U.S. Native to Asia, *A. lagerstroemiae* was first reported in 2004 in a nursery in Richardson, TX, (Dallas County) [6]. The wide distribution of crapemyrtles in the U.S. may facilitate the rapid spread of *A. lagerstroemiae*. Associated with accumulation of black sooty mold (Figure 1), *A. lagerstroemiae* infestations could cause aesthetic damage to crapemyrtle [4,7]. Because of this plant damage, *A. lagerstroemiae* was recognized as one of the top nine pests in 2015 by the Greenhouse Grower magazine [8].

Information on the biology, ecology and control of *A. lagerstroemiae* is limited, and most comes from field observations in different regions in Asia. For example, the number of generations of *A. lagerstroemiae* ranged from two to four per year depending on the location [9,10,11,12]. Management strategies have focused on chemical control including the use of cypermethrin emulsion and lime sulfur, which reported to be effective in suppressing nymphs in China [9,13]. However, information on overwintering ecology, host plant resistance, and biological control remains unknown. The objectives of this review are to present current knowledge about the biology and ecology of *A. lagerstroemiae*, and to suggest research approaches for implementing integrated pest management (IPM) programs focusing on pest’s thermal tolerance, host range, and the evaluation for host plant resistance and biological control.

## 2. Taxonomy

*Acanthococcus lagerstroemiae* (Kuwana), formerly *Eriococcus lagerstroemiae* Kuwana (Hemiptera: Eriococcidae), was combined into the genus *Acanthococcus* (Acanthococcidae) in 2013 along with 345 other species [14]. The family Eriococcidae sensu lato contained about 80 species in 10 genera in the U.S. [15], with some important ornamental plant scale pests. Kozar et al. (2013) placed most of these scales into Acanthococcidae (Group Family), for example, *Acanthococcus* (=*Eriococcus*) *azalea* (Comstock) on azaleas and *Gossyparia spuria* (Modeer) on elms [14,16]. The definition and borderlines of the family Eriococcidae (or Acanthococcidae) is still debated among coccidologists due to its diverse morphology and behavior [14,15]. Molecular analyses using the nuclear small subunit ribosomal RNA gene (SSU rRNA or 18S) also suggested the polyphyletic relationships within genus *Eriococcus* sensu lato [17]. Here we refer to the crapemyrtle bark scale as *A. lagerstroemiae* based on a latest review of the genus [14,18].

## 3. Biology

**Figure 2:**
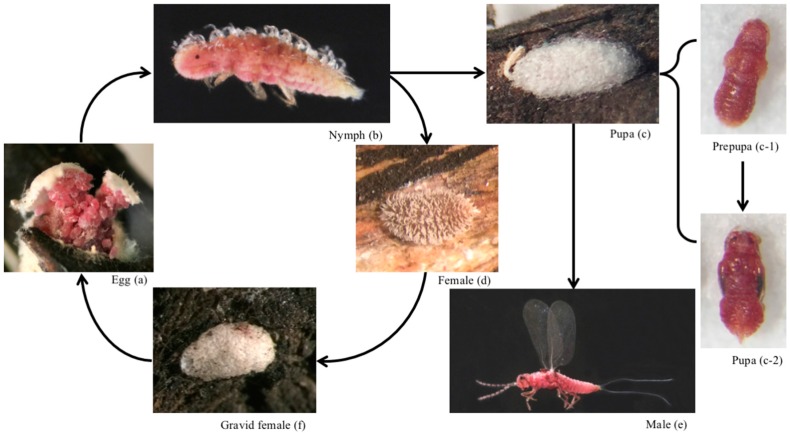
Life cycle of *Acanthococcus lagerstroemiae*: (**a**) Egg; (**b**) Nymph; (**c**) Pupa covered with white sac; (**c-1**) Prepupa; (**c-2**) Pupa; (**d**) Adult female; (**e**) Adult male; and (**f**) Ovisac containing the gravid adult female.

*Acanthococcus lagerstroemiae* has the same incomplete metamorphosis as other species in the superfamily Coccoidea [19]. The female is paedomorphic, meaning that its form resembles that of a nymph [4,7]. The male turns into an alate without mouthparts after the prepupal and the pupal stage [7]. Eggs are 0.35 ± 0.05 mm (mean ± standard error) long, 0.15 ± 0.05 mm wide (n = 20), pink, and surrounded with white filaments (Figure 2a). Eggs are laid inside the white felt-like covering secreted by the female.

Nymphs are pink and mobile (Figure 2b). The first instars or crawlers are 0.5 ± 0.1 mm long and 0.15 ± 0.05 mm wide (n = 20). After hatching, crawlers settle on the woody parts of the stem and new growth. Three nymphal stages were observed [7]. Nymphs and females secrete honeydew as a result of feeding.

Male pre-pupae and pupae are pink, non-feeding, immobile, and completely enclosed by white sacs (Figure 2c). Male pre-pupae are 0.9 ± 0.1 mm long, 0.4 ± 0.1 mm wide (n = 20), and male pupae are 1.2 ± 0.1 mm long and 0.5 ± 0.1 mm wide (n = 20) (Figure 2c-1). The blackish eyes and wing pads in the pupal stage are distinct from the pre-pupae (Figure 2c-2). Males are pink, alate, and have two long white filaments at the tip of the abdomen (Figure 2e). The mesothoracic wings have reduced venation, and the metathoracic wings have been lost along with the mouthparts. There are two pairs of ocelli each on dorsal and ventral side of the head, and a pair of smaller lateral ocelli. The filaments and extra ocelli have also been observed on other scales in Coccoidea which might function to stabilize the flight [20].

Females are 2.0 ± 0.9 mm long, 1.2 ± 0.6 mm wide (n = 20), wingless, pink, and sessile (Figure 2d). Female shape and size varies according to the location of settling and presence of eggs inside the abdomen, but in general the size is much larger than the male. After production of the white ovisac, all eggs are laid, the female decreases in size and dies. The female white ovisac likely functions as a barrier against natural enemies and a mechanism to maintain humidity (Figure 2f).

*Acanthococcus lagerstroemiae* has high fecundity and populations can grow rapidly. Females lay from 114 to 320 eggs during their lifetime [10]. After hatching and each molting, the crawlers and later instars disperse along the branches for one to two days and then become sessile [7]. Scales colonize the leaves, branches, twigs, trunk, stems and fruits. Some empirical evidence suggests that females and males have three and five nymphal stages, respectively [7]. Number of generations per year ranges from two to four depending on the climate in Asia [9,10,11,12] and is thought to be two to four in the U.S. [4]. In Anhui, China (31°81′ N, 117°21′ E), two generations each year were observed [9], and the life cycle from egg to adult varied from 56 to 83 days [10]. In Guiyang, China (26°41′ N, 106°68′ E) and Sichuan, China (27°95′ N, 102°21′ E), four generations were recorded [11,12]. In Asia, *A. lagerstroemiae* overwinters as egg, nymph, prepupa and pupa [9,11,21], while in the U.S., it has been reported to overwinter as nymphs [4].

## 4. Host Range

Host records revealed that *A. lagerstroemiae* not only attacks crapemyrtle but also other plant species in different families. In China, Japan, and Korea, this pest has been reported on thirteen other plants of ecological and economic importance (Table 1). For example, *A. lagerstroemiae* was reported to be a problem to pomegranate, *Punica granatum* L. (Myrtales: Lythraceae) in Pan Xi District, Sichuan, China (27°02′ N, 101°44′ E), due to sooty mold accumulation [12]. Despite being present in the U.S. for more than ten years, *A. lagerstroemiae* has only been reported feeding on crapemyrtle [4] and American beautyberry (*Callicarpa americana* L.) [22]. Understanding the impact of *A. lagerstroemiae* to other plant species in the U.S. could help predict the potential economic damage and prevent its spread to other plant species.

## 5. Distribution and Dispersal

**Figure 3:**
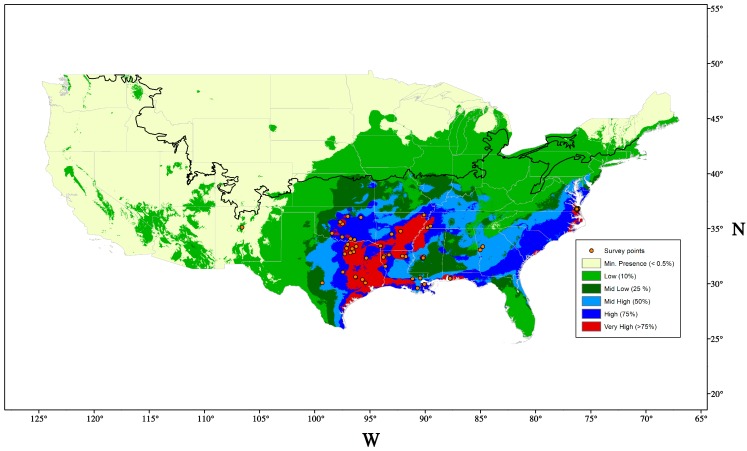
Projected distribution of *Acanthococcus lagerstroemiae* in the U.S. Warmer color indicates the higher climatic suitability. Orange points indicate the location of reported infestation. The black line indicates the upper limit of Plant Hardiness Zone 6.

*Acanthococcus lagerstroemiae* is widely distributed in Asia. The most northern and southern locations reported in Asia are Beijing, China (40°12′ N, 116°21′ E) [29] and Tamil Nadu, India (10°77′ N, 78°71′ E) [30], respectively. It was reported in England [23] in 1915 in a nursery but has not been reported since then [31]. Since its first detection in 2004 [4], *A*. *lagerstroemiae* has been reported in the U.S. states of Alabama, Arkansas, Georgia, Louisiana, Mississippi, New Mexico, North Carolina, Oklahoma, Tennessee, Texas, Virginia [32], and Washington [33], as of August, 2016. To predict the potential geographic distribution of the scale, we performed a niche modeling exercise using worldwide locations (MaxEnt version 3.3.2; http://www.cs.princeton.edu/~schapire/maxent/) [34]. Eighty-two confirmed locations were used in the model, including 57 locations in the U.S., 22 locations in China, and one location each in Japan, Korea, and India, respectively (Table S1). We used altitude and 19 bioclimatic (bioclim) variables related to temperature and precipitation from the WORLDCLIM database (http://www.worldclim.org) to predict the climatic suitability in the U.S. and Asia. The prediction suggested that *A. lagerstroemiae* has established in different climates, and perhaps has reached the upper geographical limit in the U.S. (Figure 3).

Short distance dispersal of *A. lagerstroemiae* occurs by nymphs, and long-distance dispersal could be attributed to wind, birds, and human activities [4]. Morphological characters of crawlers could facilitate its dispersal by wind including flat and small body, relatively long legs, and lateral wax filaments on the body fringe [20]. Under experimental conditions, birds were capable of transferring nymphs of the hemlock woolly adelgid, *Adelges tsugae* (Annand) (Hemiptera: Adelgidae) by touching infested branches [35]. Crawlers of four armored scales, including *Aspidiotus nerii* Bouche (Hemiptera: Diaspididae), *Abgrallaspis aguacatae* Evans, Watson & Miller, *Hemiberlesia lataniae* (Signoret), and *Diaspidiotus perniciosus* (Comstock), were found possessing a suction cup-like structure on hairs at the end of each leg, which can help them latch on larger insects to disperse [36]. We suspect *A. lagerstroemiae* could use larger animals to disperse. The discontinuous reports of *A. lagerstroemiae* in the U.S. (Figure 3. Survey points) suggested that human activities, trade and transportation of infested crapemyrtles could have facilitated the pest’s long-distance movement. Measures should be taken to prevent further dispersal, for example, sales-stop restriction in reported area [37]. Potential distribution range estimated by climatic suitability and host range can help early detection and timely management.

## 6. Plant Damage and Economic Impact

*Acanthococcus lagerstroemiae* does cause significant damage to its host plant. Several instances suggested heavy infestation of *A. lagerstroemiae* could cause branch dieback (Figure 2) and stunt growth [10,11,12]. Limited empirical evidence has suggested a reduction in blossoms as a result of infestation with *A. lagerstroemiae* [38]. The scale secretes honeydew, which facilitates the growth of black sooty mold [4,10,11,12,39] and could interfere with plant photosynthesis; in addition, the coverage of ovisacs in the truck and branches is aesthetically displeasing. Extensive honeydew deposits and sooty mold can turn branches and trunks to an unappealing black color, significantly reducing landscape aesthetic value of infested plants [4]. However, relationship between population density of *A. lagerstroemiae* and different aspects of plant damage is still unclear. Research on this relationship may provide decision-making guidance on management options.

The economic impact of *A. lagerstroemiae* has not been quantified. However, failure to manage this exotic pest could lead to serious economic loss for wholesale and retail nurseries, landscape professionals, and consumers. To manage *A. lagerstroemiae*, nurseries would have to increase labor and insecticides which could result in greater costs [4]. This scale could also potentially decrease the production and market value of crapemyrtle because of reduced sales. In states such as Arkansas, Louisiana, Oklahoma, Tennessee, and Texas, the stop-sale restriction of crapemyrtle has been enacted in nurseries with *A. lagerstroemiae* infestation [37]. Because some of the potential hosts of *A. lagerstroemiae* are fruit crops of economic importance, for example, paradise apple, Japanese persimmon, pomegranate, fig, and brambles (Table 1), research to confirm host status of *A. lagerstroemiae* on these crops in the U.S. is critical for establishing preventive management measures.

## 7. Natural Enemies

Natural enemies of *A. lagerstroemiae* found in Asia and North America include predators and parasitoids. In Asia, the scale is attacked by the parasitoids *Grandiclavula spatulata* Zhang & Huang [40], *Metaphycus eriococci* (Timberlake), *Metaphycus cylindricus* Wang, Li & Zhang [41], *Comperiella* sp., *Clausenia* sp. [10], *Metaphycus maculatus* Agarwal [42] and *Adelencyrtus longiclavatus* Hayat, Alam and Agarwal [43] (Hymenoptera: Encyrtidae); and the predators *Chilocorus kuwanae* (Silvestri), *Chilocorus rubidus* Hope, *Rodolia limbata* Motschinsky, *Propylaea japonica* (Thunberg), *Harmonia axyridis* (Pallas) (Coleoptera: Coccinellidae), *Chrysopa septempunctata* Wesmael, and *Chrysopa sinica* (Tjeder) (Neuroptera: Chrysopidae) [10], and *Cybocephalus nipponicus* Endrody-Younga (Coleoptera: Coccinellidae) [44]. *Chilocorus kuwanae* was introduced to the U.S. from Korea in 1984 to help control euonymus scale, *Unaspis euonymi* (Comstock) (Hemiptera: Diaspididae), and was established in temperate regions (USDA Zone 7 or colder) of the U.S. after multiple releases [45].

**Figure 4:**
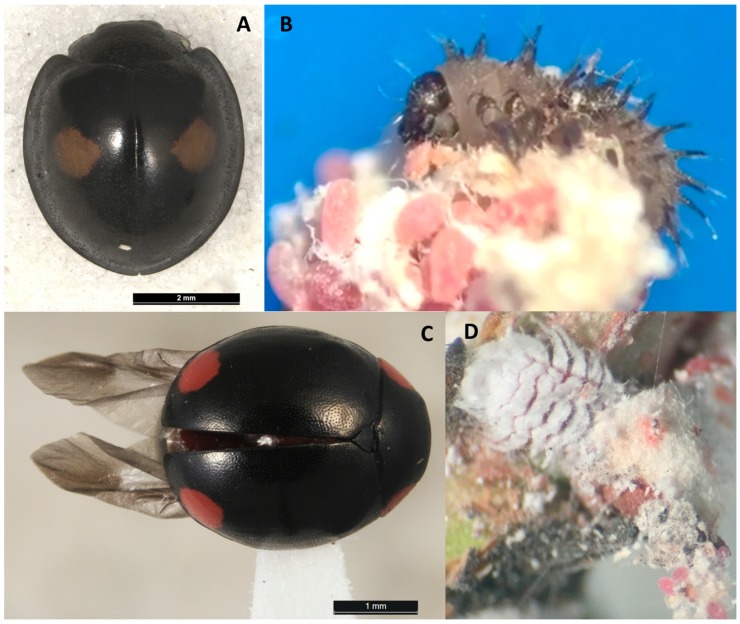
Predators of *Acanthococcus lagerstroemiae* found in Louisiana: (**A**) Adult of *Chilocorus cacti*; (**B**) Larva of *Chilocorus cacti* feeding on eggs of *Acanthococcus lagerstroemiae*; (**C**) Adult of *Hyperaspis bigeminata*; and (**D**) Larva of *Hyperaspis bigeminata* feeding on eggs of *Acanthococcus lagerstroemiae*. Voucher specimens of these two ladybeetles were deposited in Louisiana State Arthropod Museum at Louisiana State University.

**Figure 5:**
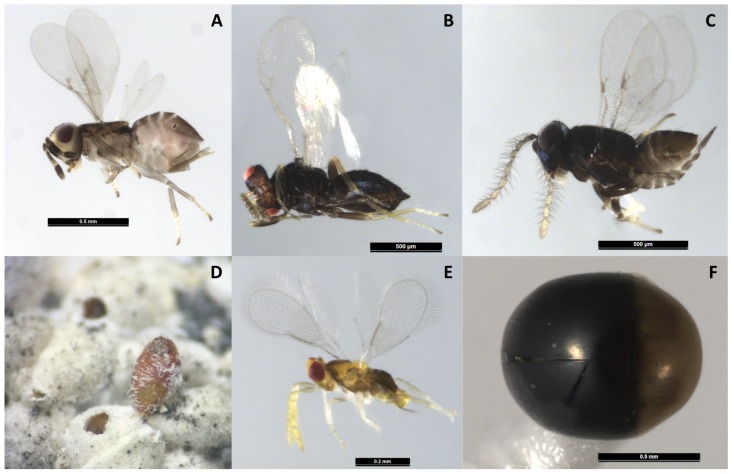
Parasitoids reared from *Acanthococcus lagerstroemiae* (**A**–**C**); caused damage in Beijing, China (**D**); the parasitoid reared in Louisiana, U.S. (**E**); and the predator, *Cybocephalus nipponicus* Endrody-Younga, reared from *Acanthococcus lagerstroemiae* in China (**F**). Voucher specimens of these natural enemies were deposited in Louisiana State Arthropod Museum at Louisiana State University.

In Louisiana, four ladybeetles (Coleoptera: Coccinellidae) were found associated with the infestation of *A. lagerstroemiae*, including two species of twice-stabbed lady beetle, *Chilocorus cacti* L. (Figure 4A,B) and *Chilocorus stigma* (Say), *Hyperaspis bigeminata* (Randall) (Figure 4C), and multicolored Asian ladybeetle, *Harmonia axyridis* (Pallas) [7]. In Texas, the ladybeetle, *Hyperaspis lateralis* Mulsant (Coleoptera: Coccinellidae) were observed in association with *A. lagerstroemiae* [46]. Field and laboratory observations in Louisiana further confirmed the predation of the cactus lady beetle, *C. cacti* and *H. bigeminata* on *A. lagerstroemiae* [47] (Figure 4B,D). We collected field samples of *A. lagerstroemiae* nymphs in several locations in Beijing, China, during the summer and also in Louisiana during the fall of 2015. Three species of unidentified Hymenopteran parasitoids were reared from females in Beijing (Figure 5A–D), and one species from nymphs in Louisiana (Figure 5E). As these parasitoids have potential to be used in classical or augmentative biological control, their morphological and molecular identification need to be confirmed. In addition, a small predacious beetle *C. nipponicus* was reared from the colony of *A. lagerstroemiae* in Beijing, China in summer 2015 (Figure 5F).

*Chilocorus cacti* is a predator of eggs and crawlers of *A. lagerstroemiae* in Louisiana and Texas (Figure 4B) [47]. In the laboratory, fourth instar of *C. cacti* can feed on about 400 scale eggs over 24 h [47]. *Chilocorus cacti* has been used as a biological control agent for several scale pests. In 1966, this predator was introduced into South Africa from Texas to control the California red scale, *Aonidiella aurantii* (Maskell) (Hemiptera: Diaspididae) [48]. Despite high predation levels and widespread releases, *C. cacti* established only in southwestern South Africa and failed to control *A. aurantii*, probably because of the extensive parasitism of *C. cacti* [49]. From 1987 to 1992, hundreds of *C. cacti* were released with other predators to control *H. lataniae* on kiwifruits, *Actinidia deliciosa* (A. Chev.) Liang et Ferguson (Ericales: Actinidiaceae) in New Zealand [50]. However, it failed to establish probably due to habitat destruction and pesticide use [51]. More research is needed to determine the potential of *C. cacti* as biological control agent for *A. lagerstroemiae*.

## 8. Current Management

*Acanthococcus lagerstroemiae* is currently managed using chemical and/or mechanical methods in the U.S. The protective covering secreted by *A. lagerstroemiae* and its feeding behavior under bark crevices make control by contact insecticides difficult [4]. In China, lime sulfur, imidacloprid, cypermethrin, methidathion, dimethoate, abamectin, triazophos, and acetamiprid have been evaluated for controlling nymphs over one generation [9,13,52,53]. However, there is no information on the efficacy of these chemicals over more generations or subsequent years. Physical methods to reduce *A. lagerstroemiae* populations include brushing infested trunks with mild dishwashing solution, and removing scales and sooty mold with high water pressure washes [4,6,39,54]. Chemical control with soil-applied systemic neonicotinoids, such as dinotefuran and imidacloprid, are most effective [4]. Adding insect growth regulator or ultrafine oils as tank-mix or rotation partners may help with long-term control. Cost of chemical control is about $10 per 10-foot-tall tree using a rotation between two neonicotinoid insecticides as estimated by Bruce Nelms, ground manager of Louisiana State University Shreveport campus, who has been treating >100 infested crapemyrtles from 2013 to 2015 [55]. Negative impacts to pollinators and natural enemies may be a concern when applying these insecticides.

## 9. Research Needed to Manage *A. lagerstroemiae*

Currently, the only options to manage *A. lagerstroemiae* are using insecticides and/or mechanical methods. Resurgence of scale densities has been observed several times at the Hammond Research Station, Hammond, LA, after treatments of systemic insecticides at recommended rates [56]. Furthermore, insecticides including imidacloprid or cypermethrin have negative non-target effects on some invertebrates including pollinators and natural enemies [57]. An integrated strategy for managing *A. lagerstroemiae* should include preventative approaches and control methods with least ecological impact. The latter part of this review provides approaches to improve the integrated management of *A. lagerstroemiae*.

### 9.1. Potential Distribution and Host Range

There is a need to study *A. lagerstroemiae* thermal tolerance to understand the pest’s phenology and its potential distribution in the U.S. Survival to temperature extremes is critical for the establishment and colonization of insects [58,59]. Mortality caused by cold and heat helps determine habitat suitability by a better understanding of the overwintering ability and heat-tolerance of exotic pests. For example, effects of cold temperatures to *Microtheca ochroloma* Stål (Coleoptera: Chrysomelidae) were studied by exposing different life stages of this chrysomelid to low temperatures for various time periods [60]. Upon the arrival of an exotic pest, thermal tolerance parameters can be useful for regulatory purposes to predict the potential distribution of the new pest in the adventive range. For example, cold tolerance of the invasive light brown apple moth, *Epiphyas postvittana* (Walker) (Lepidoptera: Tortricidae), first discovered in California in 2006, has been used to predict its potential geographic range [61]. Effects of low temperatures on mortality and oviposition of the root weevil, *Diaprepes abbreviatus* (L.) (Coleoptera: Curculionidae) were assessed to predict its future spread and area for searching potential natural enemies [62]. Similarly, knowledge of the thermal tolerance of *A. lagerstroemiae* could help predict its potential distribution in the U.S., thus providing geographic background for further research on its management.

Understanding plant species at risk of *A. lagerstroemiae* and estimating risks on host plants are critical for determining the pest’s potential spread and economic impact. Multiple plant species in different families have been reported as hosts of *A. lagerstroemiae* in its native range, but most hosts present in the literature were solely derived from unconfirmed observations. Some reported hosts are ecologically and/or economically important to the U.S., for example, pomegranate production comprises more than 30,000 acres with a $115 million value in Kern County, California alone [63]. Considering the potential and known economic values of horticultural and agronomic crops reported as alternative hosts in Asia, it is critical to evaluate all potential host plants at risk in the U.S. By using a centrifugal phylogenetic method [64], we can assess the ability of *A. lagerstroemiae* to develop (from crawlers to adult) and reproduce on plant species [65,66] closely related to crapemyrtle or those that have been reported as alternative host plants [67]. Since adult females are sessile on the host plant, no-choice experiments and life-table analysis can help compare the scale’s development, survival, reproduction and preference on selected plant species, as it has been conducted with other scales (Ex. *Tectococcus ovatus* Hempel (Hemiptera: Eriococcidae) [68]). With the host range information and preventative approaches, we can reduce or avoid economic losses to non-crapemyrtle hosts of *A. lagerstroemiae* in the U.S.

### 9.2. Plant Resistance

Host plant resistance is critical for developing IPM programs. Currently there is no published literature on crapemyrtle cultivar resistance to *A. lagerstroemiae*. With over 200 registered crapemyrtle cultivars and more than 100 cultivars commercially available in the U.S. [2], research should be conducted to study antibiosis and antixenosis of these cultivars to *A. lagerstroemiae*, which are adverse effects on the pest’s biology and behavior, respectively [69]. Tolerance can be measured for crapemyrtle cultivars by documenting a significant decrease in flowering or growth compared to others.

Integration of plant resistance into an IPM program involves screening, breeding and implementation of cultivars [69]. Resistant genotypes of crapemyrtle to survival, growth and reproduction of *A. lagerstroemiae* can be screened under no-choice condition. Crapemyrtle cultivars demonstrating resistance to *A. lagerstroemiae* can be utilized as parents in breeding. For example, daily fecundity of crapemyrtle aphids on seven crapemyrtle cultivars were screened under no-choice condition, and *L. indica* was found to have higher resistance than *L. fauriei* and *L. indica* X *fauriei* hybrids [70]. After screening 12 crapemyrtle cultivars, *L. indica* X *fauriei* hybrids with less mineral nutrient content in the leaves were less preferred by the flea beetle, *Altica litigate* Fall (Coleoptera: Chrysomelidae) [71]. At the Crapemyrtle Trails of McKinney and surrounding parks in McKinney, TX, there are more than 100 cultivars of crapemyrtles [72], providing sources for screening resistant cultivars. Scientists at the University of Florida and Texas A&M AgriLife Research and Extension Center began screening of these crapemyrtle cultivars in 2014. Current molecular technologies can help understand mechanisms underlying resistant varieties and apply this resistance to help develop new cultivars. Genomic sequencing and transcriptomic analysis can identify genes with specific resistant traits, and the virus-induced gene silencing technologies can ultimately assign resistant functions of these genes to plants [73]. Crapemyrtle cultivars with resistance to *A. lagerstroemiae* can be utilized in landscapes and evaluated for efficacy and other control strategies.

### 9.3. Biological Control

Biological control could have higher benefit/cost ratio compared with chemical and/or mechanical control strategies in terms of reduced continuous expenditure of pesticides and labor, low impacts to beneficial insects, and low risks of pest resistance [74,75,76]. Chemical or mechanical methods to control *A. lagerstroemiae* could become cost prohibitive or labor-intensive for homeowners and nursery growers. A survey of socio-economic impact of the biological control of the mango mealybug, *Rastrococcus invadens* Williams (Hemiptera: Pseudococcidae), in Benin showed that after failed trials using both mechanical and chemical controls, the release of two parasitoids have successfully controlled the mealybug with a benefit/cost ratio of 145:1 [77]. Moreover, there is a long history of successful implementation of biological control programs against scale insects. For example, three parasitoids including *Acerophagus papayae* Noyes and Schauff, *Pseudleptomastix mexicana* Noyes and Schauff, and *Anagyrus loecki* Noyes and Menezes (Hymenoptera: Encyrtidae) were introduced to India in 2010 and successfully controlled the papaya mealybug *Paracoccus marginatus* Williams and Granara (Hemiptera: Pseudococcidae), which led to an estimated net benefit between $524 million to $1.34 billion over five years [78]. In 1995, several natural enemies, including *Anagyrus kamali* Moursi (Hymenoptera: Encyrtidae) and *Cryptolaemus montrouzieri* Mulsant (Coleoptera: Coccinellidae) were introduced and have since successfully reduced the population density of the pink hibiscus mealybug, *Maconellicoccus hirsutus* (Green) (Hemiptera: Pseudococcidae) in many areas of the Caribbean [79]. The estimated net benefit of the introduction in only Trinidad was $41 million representing a socio-economic benefit/cost ratio of 8:1 for the period 1996–2024 [79]. Therefore, researchers should investigate the possibility of a biological control program for management of *A. lagerstroemiae* in the U.S. using classical, augmentative, and conservation biological control.

#### 9.3.1. Classical Biological Control

The goal of classical biological control is to introduce natural enemies from the native area to reduce pest’s populations in the adventive range. A classical biological control program involves the exploration, identification, importation, host range testing in quarantine, release and evaluation of natural enemies against *A. lagerstroemiae* in the introduced range [75].

**Figure 6:**
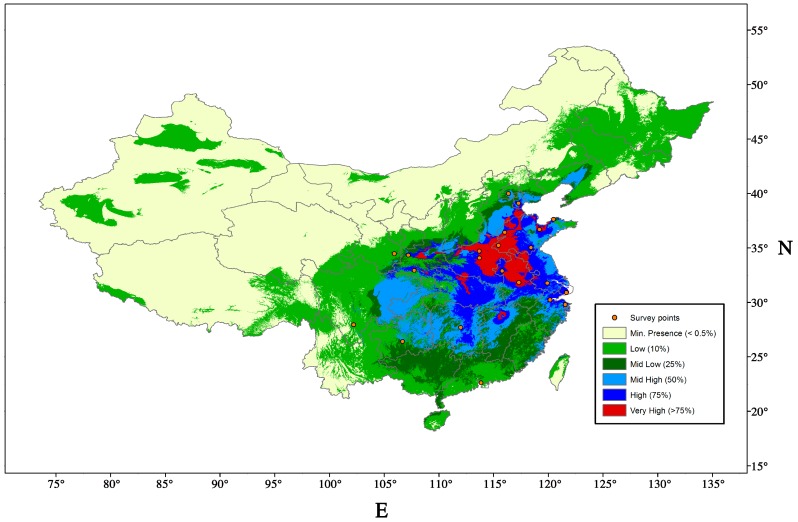
Projected distribution of *Acanthococcus lagerstroemiae* in China using MaxEnt. Warmer color indicates the higher climatic suitability. Orange points indicate the location of reported infestation.

Regions between Beijing and Jiangsu in China are ideal for exploration of natural enemies of *A. lagerstroemiae*. Based on previous climatic modeling of MaxEnt and USDA Plant Hardiness Zone Map, the northern distribution of the scale could be limited by winter temperatures and the distribution of crapemyrtle [80]. Regions with similar plant hardiness zones such as Jiangsu in China (Figure 6), Texas, and Louisiana in the U.S. (Figure 3), have very high climatic suitability (>75%) for the survival of *A. lagerstroemiae* (Figure 6). Natural enemies adapted to colder winters can be explored in the region of Beijing. In 2015, a collaboration was established with Beijing Forestry University that would facilitate long-term explorations for natural enemies in China.

Highly specialized parasitoids and predators should be prioritized in a classical biological control program of *A. lagerstroemiae* considering its effectiveness in scale control. Parasitoids with a narrower host range pose less risk to the ecosystem than other natural enemies [81]. For example, the parasitoid *Anagyrus* sp. nov. nr. *sinope* Noyes & Menezes demonstrated traits as potential biological control agents; and its highly specific to the cassava mealybug, *Phenacoccus madeirensis* Green (Hemiptera: Pseudococcidae), a pest attacking cassava, pineapple, citrus and potatoes [82]. Specialized predators are also good candidates for classical biological control programs. The vedalia beetle, *Rodolia cardinalis* (Mulsant) (Coleoptera: Coccinellidae) reduced the densities of the cottony cushion scale, *Icerya purchasi* Maskell (Hemiptera: Monophlebidae) [83], though this beetle’s host range was determined to be exclusively cottony cushion scales only after their introduction [84]. For natural enemies already reared from Asia, more research on their biology, ecology and host range are needed. Before introduction, the host range of potential biological control agents should be studied in a quarantine facility and tests should include native and exotic scales in the U.S. [15,16], for example, *A. azalea*, *E. quercus*, *G. spuria*, and *Eriococcus coccineus* (Cockerell) (Hemiptera: Eriococcidae).

The functional response of parasitoids to *A. lagerstroemiae* measured in quarantine can generate practical information for future field releases and mass rearing. Post-release assessment with before-and-after experimental design can evaluate the impacts of parasitoids to *A. lagerstroemiae* in the field. Comparison before and after releasing three introduced encyrtids including *A. papayae*, *P. mexicana*, and *A. loecki* in classical biological control of *P. marginatus* in Tamil Nadu in 2010 showed a 9.7% reduction in the mealybug population one month after their release and 96.6% reduction after six months [85]. These assessments can also help estimate economic benefits and the costs of classical biological control programs.

#### 9.3.2. Augmentative Biological Control

The goal of augmentative biological control is to increase the numbers of local natural enemies of *A. lagerstroemiae*. *Chilocorus cacti* and *Hyperaspis* spp. are predators of *A. lagerstroemiae* present in the southern U.S. [7,46] and have potential for augmentative biological control. However, these two ladybeetles do not appear sufficient to suppress *A. lagerstroemiae* in the field, especially in October and November when temperature begins to decrease [86]. Augmentation of these two species could reduce the overall population of *A. lagerstroemiae* over a season. For example, 30 larvae of *C. montrouzieri* per plant were released in a pomelo orchard in August 2005, and 98%, 90% and 82% of populations of the citrus mealybug, *Planococcus citri* (Risso) (Hemiptera: Pseudococcidae), the striped mealybug, *Ferrisia virgata* (Cockerell) (Hemiptera: Pseudococcidae), and the spherical mealybug, *Nipaecoccus viridis* (Newstead) (Hemiptera: Pseudococcidae) were reduced, respectively [87].

To determine the potential of these two lady beetles in augmentative biological control, researchers need to understand their life history and voracity. Study of developmental time at different temperatures will enable researchers to construct population growth models. For example, temperature-dependent development of *Chilocorus bipustulatus* L. (Coleoptera: Coccinellidae) studied under seven different temperatures suggested its optimal temperature for development between 33.6 and 34.7 °C and a thermal constant for total development of 474.7 degree-days [88]. Laboratory trials showed that eggs of *A. lagerstroemiae* can support *C. cacti* and *H. bigeminata* to develop from eggs to adults [47] and population growth models can help predict quantity and timing of release for optimal control. Similar to understand impacts of parasitoids, predation by these two ladybeetles can be assessed in the laboratory and field. Life history parameters and predation can be modeled to determine the impact of natural enemies to pest population dynamics. For example, the field life-table study of *Coccus viridis* (Green) (Hemiptera: Coccidae) in coffee plantations suggested nymphs to be the critical stage for mortality, and several coccinellid predators were considered an important factor contributing to scale mortality in the field [89]. Researchers need to conduct similar studies to determine the key life stages and factors causing mortality to *A. lagerstroemiae* in the field and determine timing for release of predators.

Rearing natural enemies can be challenging, but will be critical for conducting field augmentation studies. *Chilocorus cacti* has been studied as a biological control agent for scales that can be reared on live prey including *A. nerii* [49]. Dried wasp brood was tried as artificial diet for *C. cacti* but failed to support its oviposition [90]. No information about mass rearing of *H. bigeminata* has been reported in the literature. Culturing *C. cacti* and *Hyperaspis* spp. on *A. lagerstroemiae* may lead to problems including the discontinuity of food supply and the extra cost of rearing facilities and labor [91]. Future work could explore factitious prey or artificial diets based on vertebrate protein as alternatives for a mass rearing system, demonstrated successfully for other predators. For example, *H. axyridis* can be mass reared using eggs of the Angoumois grain moth, *Sitotroga cerealella* (Olivier) (Lepidoptera: Gelechiidae) as factitious prey [91], or the mixture of chicken egg yolk, chicken liver, sugar, casein enzymatic hydrolysate, soy oil, and different salts as artificial diet [92].

#### 9.3.3. Conservation Biological Control

The goal of conservation is to enhance the performance or increase the population density of natural enemies present in the environment by improving the habitat or reducing the exposure to toxic insecticides [93]. Chemical control has an important role in suppressing pest density. When applying insecticides it is difficult to avoid residual effects on non-target organisms [94], however, measures can be taken to minimize detrimental effects. Currently, little is known about the non-target impact of recommended insecticides used against *A. lagerstroemiae* on *C. cacti*, *Hyperaspis* spp., and parasitoids reared from China; and research about the rates, timing and delivery (i.e., drench vs. bark spray) of insecticide applications is needed.

Natural enemy populations can have more difficulty rebounding after a broad-spectrum pesticide application compared to pests, such as organophosphate and carbamate insecticides [95], and to chemical residues [96]. Soil-applied systemic neonicotinoid insecticides, which are currently the recommended strategy for *A. lagerstroemiae* management [7], are less likely to directly contact non-target organisms, but may impact natural enemies through food sources such as pollen and/or nectar of surrounding plants [95,97]. A single foliar spray of imidacloprid was demonstrated to affect survival, egg production and egg hatching of *C. septempunctata* in a laboratory study [98]. In addition, neonicotinoid residues translocated into crapemyrtle pollen could further damage the natural enemy population by causing mortality and altering behavior, such as soil-applied imidacloprid on a mealybug parasitoid, *Anagyrus pseudococci* (Girault) [99], the pink spotted lady beetle, *Coleomegilla maculata* (DeGeer) (Coleoptera: Coccinellidae) [100] and the green lacewing, *Chrysoperla carnea* (Stephens) (Neuroptera: Chrysopidae) [101]. Decrease of natural enemies may result in resurgence of *A. lagerstroemiae* after applying neonicotinoid insecticides.

Using lower insecticide dosages or timing insecticides to avoid application when natural enemies are most abundant or most susceptible can help reduce negative impact on biological control agents and reduce development of pesticide resistance [95,96,102]. The application of dormant oil when nymphs lack wax coverage should be evaluated. Studies on the interactions between insecticides and natural enemies on *A. lagerstroemiae* populations are needed. For example, low dosage of the selective aphicide pymetrozine combined with two biological control agents, the seven-spotted lady beetle, *Coccinella septempunctata* L. (Coleoptera: Coccinellidae) and *Diaeretiella rapae* M’Intosh (Hymenoptera: Aphidiidae), reduced the cabbage aphid, *Brevicoryne brassicae* L. (Hemiptera: Aphididae) population by 98% in the laboratory [103]. Negative impacts of insecticides on natural enemies can be minimized by better timing applications when populations of natural enemies are absent, and/or in life stages most resistant to the insecticides [96,102,104]. Application in winter and early spring might reduce *A. lagerstroemiae* nymphs lacking coverings without substantially impacting beneficial insects and foliar sprays can help to deliver the products considering the low sap pressure of plants during winter. To maximize survival, natural enemies should be released when insecticide residues have declined [95].

## 10. Conclusions

A successful integrated pest management strategy of *A. lagerstroemiae* requires knowledge of the scale’s biology and ecology, host range and damage to the host plant. Use of crapemyrtle varieties with high resistance to *A. lagerstroemiae* in the landscape can help improve pest management. Natural enemies may play an important role in the management of *A. lagerstroemiae* in the field and knowledge of the biology and ecology of natural enemies is needed. Classical biological control shows promise and can be started by searching for parasitoids of *A. lagerstroemiae* in regions between Beijing and Jiangsu in China. The potential for an augmentative biological control program should also be studied by investigating the efficiency of local arthropod predators in the U.S., including *C. cacti* and *H. bigeminata*, to manage *A. lagerstroemiae* populations. In addition, conservation biological control programs that include the use of narrow-range insecticides with minimal risks, reduced application rates and better timing of applications can improve IPM programs against *A. lagerstroemiae*.

## Figures and Tables

**Table 1:** Host plants of *Acanthococcus lagerstroemiae* in Asia and Hungary (except for *Lagerstroemia* spp.).

Scientific Name	Common Name	Order	Family	Country	Reference
*Anogeissus latifolia* (Roxb. ex DC.) Wall. ex Guill. & Perr.	Axlewood	Myrtales	Combretaceae	Korea	[23]
*Anogeissus* sp.	−	Myrtales	Combretaceae	China	[24]
*Buxus microphylla* Sieb. et Zucc.	Korean Boxwood	Buxales	Buxaceae	Korea	[25]
*Celtis sinensis* Pers.	Chinese hackberry	Rosales	Cannabaceae	Korea	[25]
*Dalbergia eremicola* Polhill	Indian rosewood	Fabales	Fabaceae	Korea	[23]
*Diospyros kaki* Thunb.	Japanese persimmon	Ericales	Ebenaceae	Korea	[25,26]
*Ficus carica* L.	Edible fig	Rosales	Moraceae	Korea	[25]
*Glochidion puberum* (L.) Hutch	Needlebush	Malpighiales	Euphorbiaceae	China	[27]
*Glycine max* (L.) Merr.	Soybean	Fabales	Fabaceae	China	[27]
*Ligustrum obtusifolium* Sieb. et. Zucc.	Border privet	Lamiales	Oleaceae	−	[14]
*Malus pumila* Mill.	Paradise apple	Rosales	Rosaceae	China	[27]
*Mallotus japonicus* Muell. Arg.	Food wrapper plant	Malpighiales	Euphorbiaceae	Korea	[25,28]
*Myrtus* sp.	Myrtle	Myrtales	Myrtus	Hungary	[14]
*Punica granatum* L.	Pomegranate	Myrtales	Lythraceae	China and Korea	[25,27,28]
*Pseudocydonia sinensis* Schneid.	Chinese-quince	Rosales	Rosaceae	Korea	[26]
*Rubus* sp.	Brambles	Rosales	Rosaceae	Hungary	[14]

## Supplemental Materials

The following are available online at http://www.mdpi.com/2075-4450/7/4/78/s1, Table S1: Occurrence of infestation of *Acanthococcus lagerstroemiae* in the U.S. and Asia.
