# Supplementary material for: Crapemyrtle Bark Scale: A New Threat for Crapemyrtles, a Popular Landscape Plant in the U.S
Source: Insects. 2016 Dec 16;7(4):78. doi: 10.3390/insects7040078 (PMC5198226; doi:10.3390/insects7040078)
Supplement: Supplementary file 1 [file insects-07-00078-s001.pdf]

Supplementary Materials: Crapemyrtle Bark Scale: A New Threat for Crapemyrtles, a Popular Landscape Plant in the US

Zinan Wang, Yan Chen, Mengmeng Gu, Erfan Vafaie, Michael Merchant and Rodrigo Diaz

Table S1. Occurrence data of *Acanthococcus lagerstroemiae* (Kuwana) (Hemiptera: Eriococcidae). Occurrence data of 57 coordinates in USA were retrieved from EDDMapS (Early Detection and Distribution Mapping System) (https://www.eddmaps.org/). Occurrence data of 25 coordinates in Asia were retrieved from local reports and personal communications. Coordinates were obtained using Google Map (http://maps.google.com/) for reports with detailed address. For infestation reports only with vague description, a point in the center of the described polygon was chosen.

Reports in USA	Longitude	Latitude
1	−87.756827	30.476208
2	−90.1148	36.2327
3	−92.237298	34.766021
4	−93.050627	34.49612
5	−94.034167	33.434928
6	−84.798882	33.374325
7	−85.019812	33.075882
8	−90.072656	29.950249
9	−90.465941	30.516369
10	−90.712452	29.595381
11	−91.754314	32.474734
12	−92.08057	32.51462
13	−93.28371	32.619825
14	−93.718015	32.526937
15	−93.785576	32.400182
16	−93.5957	31.583
17	−90.228713	32.298431
18	−90.109288	32.456571
19	−90.181246	32.35141
20	−106.605068	35.118083
21	−95.8386	36.0209
22	−97.0168	36.116
23	−97.506663	35.43287
24	−97.7199	35.6058
25	−98.4454	34.5865
26	−96.3976	34.007
27	−96.7753	34.0903
28	−97.528715	34.248939
29	−89.807391	35.085743
30	−89.519472	35.225619
31	−94.062628	33.440702
32	−96.247443	33.379875
33	−97.018053	33.571722
34	−97.129359	33.209732
35	−96.612093	33.172243
36	−99.337492	30.070782
37	−97.284618	32.713096
38	−96.784898	32.80162

39	−96.464699	32.89801
40	−97.470737	31.052155
41	−95.337972	32.338723
42	−96.2987	30.5983
43	−95.698205	30.389004
44	−95.416388	30.076222
45	−96.321863	30.623193
46	−76.26711	36.7762
47	−76.294734	36.72543
48	−76.127973	36.843111
49	−76.26281	36.776564
50	−76.225156	36.657395
51	−76.282046	36.719318
52	−76.295063	36.721198
53	−76.330052	36.727818
54	−76.300819	36.861877
55	−76.197416	36.811324
56	−76.193157	36.76864
57	−76.26711	36.7762
**Reports in Asia**	**Longitude**	**Latitude**
1	78.718094	10.768851
2	135.77125	35.009334
3	128.610432	35.888726
4	121.652112	30.913046
5	116.347888	40.002954
6	102.209878	27.945995
7	117.271727	39.098239
8	115.786498	32.893185
9	117.294159	31.836276
10	116.005074	36.427981
11	115.445945	35.240412
12	118.416813	35.031681
13	119.180717	36.694145
14	120.473516	37.631029
15	113.846879	22.622914
16	120.150914	30.242408
17	119.898736	31.781352
18	113.68309	34.715524
19	113.697929	34.088935
20	121.545597	29.810677
21	111.985543	27.695953
22	105.912011	34.482543
23	107.752551	32.9364
24	106.677558	26.40761
25	107.196238	34.342516

© 2016 by the authors; licensee MDPI, Basel, Switzerland. This article is an open access article distributed under the terms and conditions of the Creative Commons by Attribution (CC-BY) license (http://creativecommons.org/licenses/by/4.0/).
